# A Newcastle disease outbreak in backyard and free-range poultry in Slovenia

**DOI:** 10.3389/fvets.2026.1803248

**Published:** 2026-04-07

**Authors:** Zoran Žlabravec, Brigita Slavec, Alenka Dovč, Aleksandra Hari, Joško Račnik, Jedrt Maurer Wernig, Edoardo Giussani, Elisa Palumbo, Maria Varotto, Isabella Monne, Uroš Krapež

**Affiliations:** 1 Institute for Poultry, Birds, Small Mammals, and Reptiles, Veterinary Faculty/National Veterinary Institute, University of Ljubljana, Ljubljana, Slovenia; 2 Animal Health and Welfare Division, Administration for Food Safety, Veterinary Sector and Plant Protection, Ljubljana, Slovenia; 3 European Reference Laboratory (EURL) for Avian Influenza and Newcastle Disease, Istituto Zooprofilattico Sperimentale Delle Venezie, Padua, Italy

**Keywords:** backyard poultry, free-range poultry, genotype VII.1.1, Newcastle disease, *Orthoavulavirus javaense*, Slovenia

## Abstract

Newcastle disease (ND) is a highly contagious and economically important viral disease of poultry, caused by virulent strains of *Orthoavulavirus javaense* (OAVJ). In Slovenia, vaccination is mandatory in commercial poultry and selected avian species, whereas backyard and free-range flock remain largely unregulated. In January and February 2025, two unrelated backyard and free-range flock of laying hens experienced acute outbreaks with severe clinical signs, increased mortality and distinct gross necropsy findings (haemorrhages in the mucosa of the gastrointestinal tract, multifocal pale foci in the liver, atrophy to enlarged hyperaemic spleen, oophoritis and salpingitis). OAVJ was detected in cloacal, oropharyngeal, and brain samples from both outbreaks using real-time RT-qPCR. Analysis of complete fusion (F) gene revealed identical nucleotide sequences in both outbreaks, with the cleavage site motif, characteristic of velogenic strains (amino acid sequence 112RRQKR116 at the C-terminus of the F2 protein and F at residue 117, the N-terminus of the F1 protein). Further phylogenetic analysis of the F gene demonstrated that both viruses belong to class II, sub-genotype VII.1.1, showing 99.6–100% identity with strains recently detected in Poland. The hemagglutinin-neuraminidase (HN) glycoprotein comprised 571 amino acids, consistent with genotype VII viruses, with several amino acid substitutions previously associated with functional relevance. These results highlight that, despite the absence of ND in Slovenia for more than three decades, local poultry populations remain highly susceptible to the introduction of the virus, especially in backyard flocks where monitoring and disease control are difficult to implement. Although a virulent virus was confirmed in both outbreaks, no further spread to other poultry holdings was detected.

## 1 Introduction

Newcastle disease (ND) is a devastating and highly contagious infectious disease of poultry with significant economic impact and global distribution, despite large-scale vaccine implementation (1). It is listed by the World Organization for Animal Health (WOAH) as a notifiable disease due to its potential for rapid spread and trade implications (2).

The causative agent of ND is *Orthoavulavirus javaense* (OAVJ), formerly known as avian orthoavulavirus 1 (AOAV-1), or avian paramyxovirus type 1 (APMV-1), or Newcastle disease virus (NDV), belonging to the genus *Orthoavulavirus*, subfamily *Avulavirinae*, family *Paramyxoviridae* (3). For consistency, in this work we will use the acronym OAVJ when referring to the pathogen. The virus has a negative-sense, single-stranded, non-segmented RNA genome encoding six structural proteins: nucleoprotein (NP), phosphoprotein (P), matrix protein (M), fusion protein (F), hemagglutinin-neuraminidase (HN), and large polymerase (L) (4, 5). Among these, the F protein is the major determinant of viral virulence, moreover, phylogenetic analysis of the F gene coding sequenceis primarily used to classify OAVJ viruses into genetic classes I and II, each containing multiple genotypes and sub-genotypes (6). Class II viruses are primarily involved in ND outbreaks and are subdivided into at least 21 genotypes (I–XXI) (6). In recent years, genotype VII, particularly sub-genotypes VII.1.1 and VII.2, has been predominant in Asia, Africa, and the Middle East, while genotype XIII has been reported in parts of Africa and South Asia (7–11). Genotype VI viruses are still frequently isolated from pigeons and doves in Europe (12, 13). Recent European outbreaks in 2025 include detections in several countries including Poland, the Netherlands, Malta, North Macedonia, Spain, Bulgaria and Slovakia. Reports describe both pigeon-adapted strains and strains typically detected in poultry (14, 15).

The pathotypes of OAVJ are categorized, based on virulence in chickens after experimental inoculation, into asymptomatic enteric, lentogenic, mesogenic, and velogenic strains. Velogenic strains are highly virulent and divided into viscerotropic velogenic viruses, causing haemorrhagic lesions in the gastrointestinal tract with high mortality, and neurotropic velogenic viruses, causing neurological signs with respiratory involvement (2). Clinical presentation is variable, depending on virus strain, host species, age, concurrent infections, environmental factors, and immune status (16).

ND is endemic in many regions worldwide, particularly in Africa and Asia, where vaccination coverage and biosecurity practices remain insufficient to control virus circulation (1, 16). In Europe, ND is largely controlled through compulsory vaccination programs for commercial poultry; however, the risk of outbreaks persists due to virus introductions via migratory birds, trade of live birds, and inadequate vaccination or biosecurity in non-commercial flocks (2, 14, 15).

In Slovenia, since 1993, vaccination against ND has been mandatory for flocks with more than 350 poultry (including chickens, turkeys, Japanese quails, guinea fowls), ostrich flocks, and parent flocks of pheasants and partridges, as well as for pigeons intended for exhibitions or competitions (17). However, vaccination in backyard or hobby flocks remains neither regulated nor monitored. Early detection relies on notification by owners or veterinarians of clinical signs suggestive of ND or avian influenza (AI), including sudden mortality, reduced feed and water intake, decreased egg production, respiratory or neurological signs. The last recorded outbreak of ND in Slovenia occurred in 1991 (18).

This study reports the clinical, pathological, and molecular characterization of two backyard/free-range poultry outbreaks of Newcastle disease in Slovenia in 2025, representing the first detection after more than three decades.

## 2 Materials and methods

### 2.1 Case history

**Figure 1:**
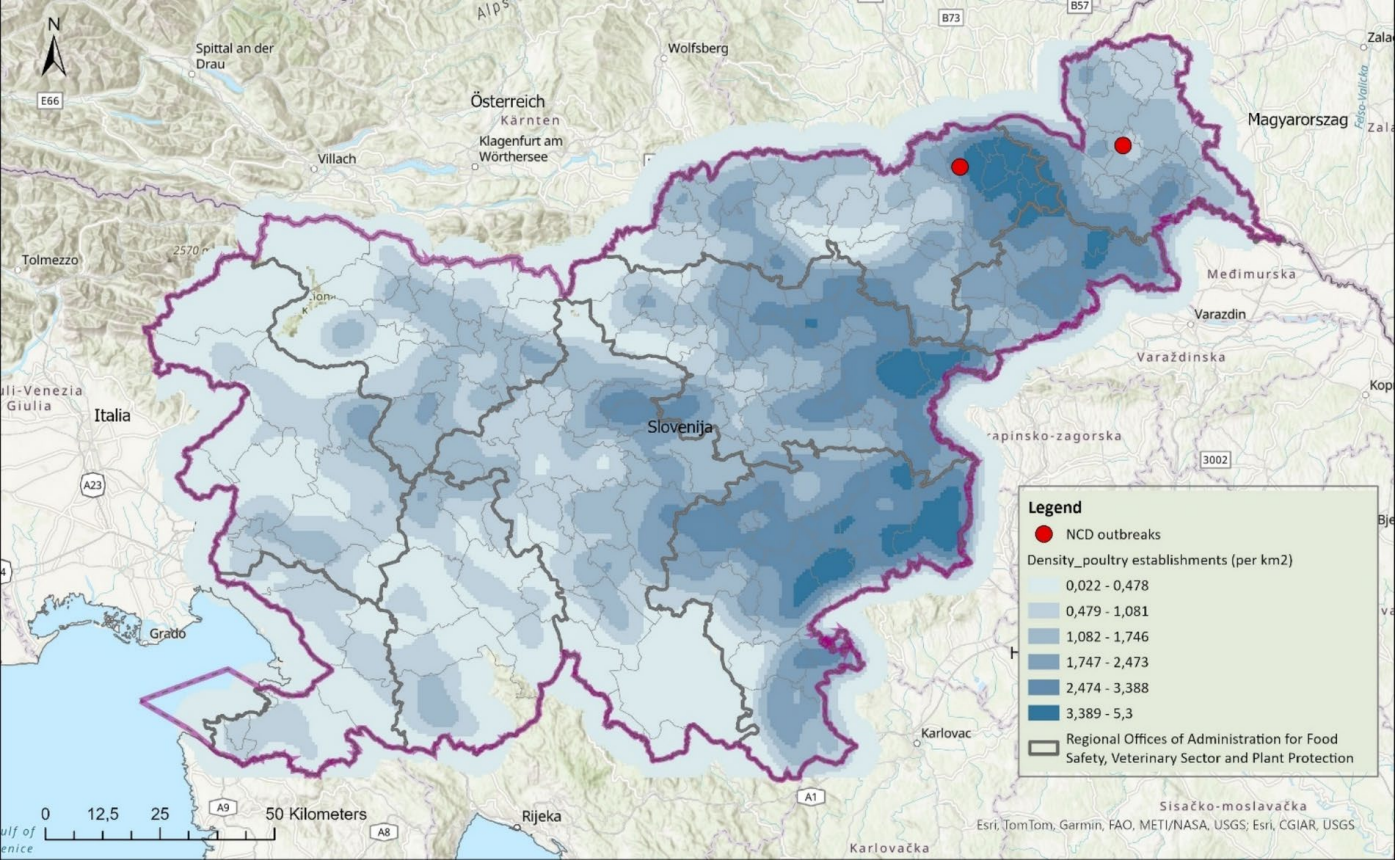
Geographic distribution of confirmed ND cases in Slovenia in 2025.

The first outbreak/case (flock 1; case number 44/25) was detected in January 2025 in a backyard flock of 14 laying hens aged 1.5 to 4 years in the municipality of Murska Sobota in north-eastern Slovenia (Figure 1). The second outbreak/case (flock 2; case number 347/25) occurred in February 2025 in a free-range flock of 160 laying hens of different ages in the municipality of Pesnica, north-eastern Slovenia. The two outbreaks were epidemiologically unrelated. In both outbreaks, clinical signs such as a sudden increase in mortality – 57% (8/14) and 73% (116/160) within five days, depression, weakness and cyanosis of the head were observed. The dead birds were handed over to the Institute of Birds, Small Mammals and Reptiles, Faculty of Veterinary Medicine/National Veterinary Institute, University of Ljubljana, for necropsy and further necessary diagnostic tests, mainly molecular diagnostics of AI and ND.

### 2.2 Pathology, histopathology, and bacteriology

The submitted dead birds were grossly examined, and tissue specimens (intestine, proventriculus, gizzard, spleen, liver, pancreas, oviduct, kidney, trachea from the first case; trachea, spleen, heart, kidney, liver, proventriculus, gizzard, oesophagus, lungs from the second case) were collected for histopathology examination. The samples were fixed in 10% buffered formalin and sent to the Institute of Pathology, Wild Animals, Fish, and Bees, Veterinary Faculty, University of Ljubljana to perform histopathology analyses. For bacteriology examination liver and spleen from first case and liver and heart from second case were sent to Institute of Microbiology and Parasitology’s, Veterinary Faculty, University of Ljubljana.

### 2.3 Molecular analyses

For molecular analyses, swabs of oropharynx (including trachea), cloaca and brain homogenate were collected from up to five dead birds per submission and pooled by sample type. Total nucleic acid was extracted from 140 μL of supernatant using the QIAamp Viral RNA Mini Kit (Qiagen, Germany). Real-time RT-PCR for detection of AI virus was performed as described previously (19). Detection of OAVJ was performed using the RT-qPCR protocol for the L gene recommended by the European Union Reference Laboratory (EURL) (20) with minor modifications. RT-qPCR assays were performed using the AgPath-ID^™^ One-Step RT-PCR Kit (Thermo Fisher Scientific, United States) on a QuantStudio^™^ 5 system.

To analyse the cleavage sites of the F gene, RT-PCR was performed using the primers described by Krapež et al. (21) and the One-Step RT-PCR Kit (Qiagen). The PCR products (~454 bp) were visualized on a 1.8% agarose gel, purified (FastGene Gel/PCR Extraction Kit, Nippon Genetics Europe) and submitted for Sanger sequencing (Eurofins Genomics, Germany). The sequences were analyzed by BLAST to determine the genotype and motif of the cleavage site.

### 2.4 Virus isolation

For virus isolation, swab supernatants were mixed with antibiotics (penicillin 5,000 IU/mL, streptomycin 5,000 μg/mL), antimycotics, and amphotericin B (12.5 μg/mL). After centrifugation at 1,000 × g for 10 min, 0.2 mL of the supernatant was inoculated into the allantoic cavity of four 10-day-old embryonated chicken eggs. Eggs were incubated at 37 °C and candled daily. Dead embryos were removed and chilled immediately; all remaining eggs were chilled at day five post-inoculation. Allantoic fluids were harvested and tested by RT-qPCR, and positive samples were used for genome sequencing.

### 2.5 Genome sequencing and phylogenetic analysis

To obtain the whole genome sequences of the two detected viruses, metagenomic sequencing was performed at the European Reference Laboratory for avian influenza & Newcastle disease (the Istituto Zooprofilattico Sperimentale delle Venezie), using RNA extracted from cloacal swabs for the virus identified in the backyard poultry farm (flock 1; case number 44/25) in the municipality of Murska Sobota and from allantoic fluid for the virus identified in the free-range flock of laying hens (flock 2; case number 347/25) located in the municipality of Pesnica. Abundant rRNA and globin RNA were removed starting from 120 ng of total RNA by the Illumina Stranded Total RNA Prep, Ligation with Ribo-Zero Plus (Illumina, San Diego, CA, United States). Ribodepleted samples then underwent library preparation and were processed on an Illumina Miseq instrument using a MiSeq Reagent Kit v2 (2 × 250 bp paired-end [PE] mode) (Illumina, San Diego, CA, United States). The consensus sequence of the whole genome was obtained using an in-house Nextflow pipeline (22). Sequencing adapters and low quality (<Q20) 3′ ends were trimmed with Cutadapt version 4.5 (23). All reads shorter than 80 bp after the trimming were discarded. Reads quality was assessed with FastQC v.0.12.1 (24). Reads were mapped with BWA 0.7.17 (25) and the obtained alignment was corrected and improved using GATK version 4.4.0.0 (26). Variant calling was performed using LoFreq v.2.1.5 (27) and the consensus sequence was generated with an in-house Python script.

The obtained sequences were analysed to reconstruct the phylogenetic relationships between the Slovenian viruses and sequences available in the public GenBank repository. The initial reference set (*n* = 128) corresponded to the NDV curated class II dataset (May 09, 2022 release), including representative full-length F gene sequences for all class II sub/genotypes defined in the study by Dimitrov et al. (6). In addition to these reference sequences, we included further GenBank sequences retrieved through BLAST searches to capture the closest publicly available relatives of the Slovenian viruses.

Multiple sequence alignments of the NDV F gene were produced in MEGA7 (28) using the MAFFT plugin (29, 30). Model selection was performed in IQTREE v1.6. (30) using ModelFinder, and the best-fit model, according to the Bayesian Information Criterion (GTR + F + R4, General Time Reversible with empirical base frequencies and FreeRate among-site rate heterogeneity with four categories), was used for maximum-likelihood tree inference with 1,000 bootstrap replicates. The software FigTree v1.4.3. (31) was used to visualize the phylogenetic tree.

## 3 Results

### 3.1 Pathology, histopathology and bacteriology findings

**Figure 2:**
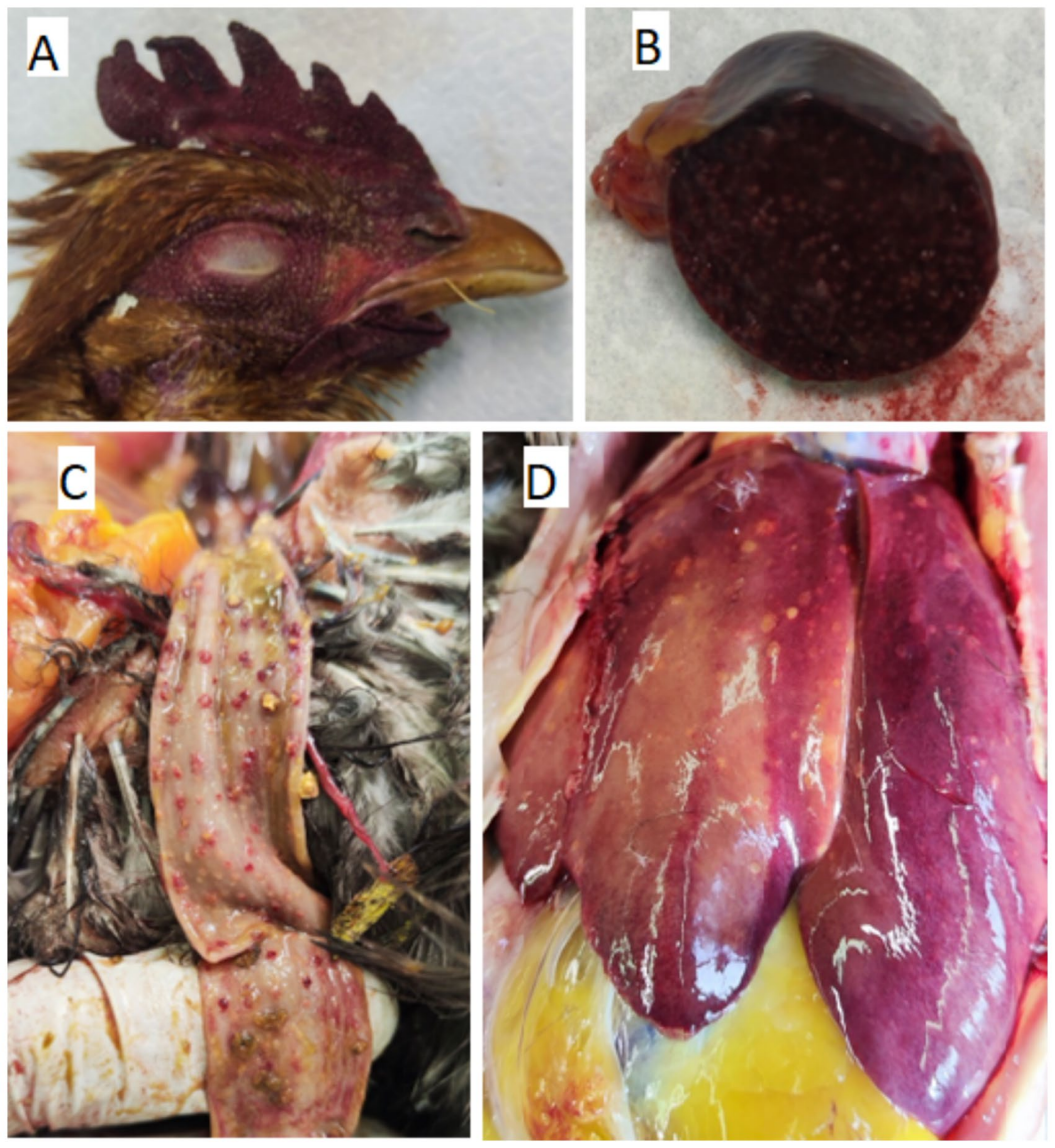
Gross pathological findings in submitted hens. **(A)** Cyanosis of the head, comb, and wattles. **(B)** Hyperemic and enlarged spleen. **(C)** Hemorrhages in the mucosa of colon **(D)** Discolored and multifocal pale foci of the liver.

At necropsy of three layers from the first case (flock 1; 44/25), cyanosis of the head, comb, and wattles was observed, together with dehydration. The tracheal mucosa was reddened, with mucoid exudate and haemorrhagic tracheas. The liver showed discoloration with multifocal pale foci, while the spleen was hyperaemic, enlarged, and diffusely mottled. Hyperaemia of the lungs and enlarged, hyperaemic kidneys were also noted. Oophoritis and salpingitis were present, characterized by accumulation of thick yellow exudate on both surfaces. Petechial to ecchymotic haemorrhages in the mucosa of the proventriculus, gizzard, jejunum, and colon were observed (Figure 2).

During routine histopathological examination, necrosis and lymphocytic infiltration were observed in the intestines, proventriculus, gizzard, spleen, liver, pancreas, kidneys, and trachea. Additionally, haemorrhage was detected in the intestinal mucosa, whereas only lymphocytic infiltration was present in the ventriculus and oviduct.

At necropsy of five layers from the second case (flock 2; 347/25), cyanosis of the head, comb, and wattles and dehydration were observed. The liver was mottled, with multifocal pale foci and petechial haemorrhages, while atrophy of the spleen was noted. Mucoid exudate was present in the tracheas, and ecchymotic haemorrhages were observed in the mucosa of the proventriculus and duodenum. Mild endoparasitosis (Ascariasis) and scaly, crusty lesions on the legs consistent with moderate mite infestation (*Knemidocoptes* sp.) were detected. Oophoritis and salpingitis were also present.

During routine histopathological examination, necrosis was observed in the trachea, spleen, heart, kidneys, proventriculus, ventriculus, and lungs. Additionally, lymphocytic infiltration was detected in the proventriculus and ventriculus, haemorrhage in the trachea, and purulent peribronchitis.

Bacteriological examination showed the presence of *Escherichia coli* in the liver and *Clostridium perfringes* in the spleen from flock 1 (44/25) and isolated colonies of *Pasteurella multocida* and *Gallibacterium anatis* in the liver and heart from flock 2 (347/25).

### 3.2 Molecular and phylogenetic characterization

**Figure 3:**
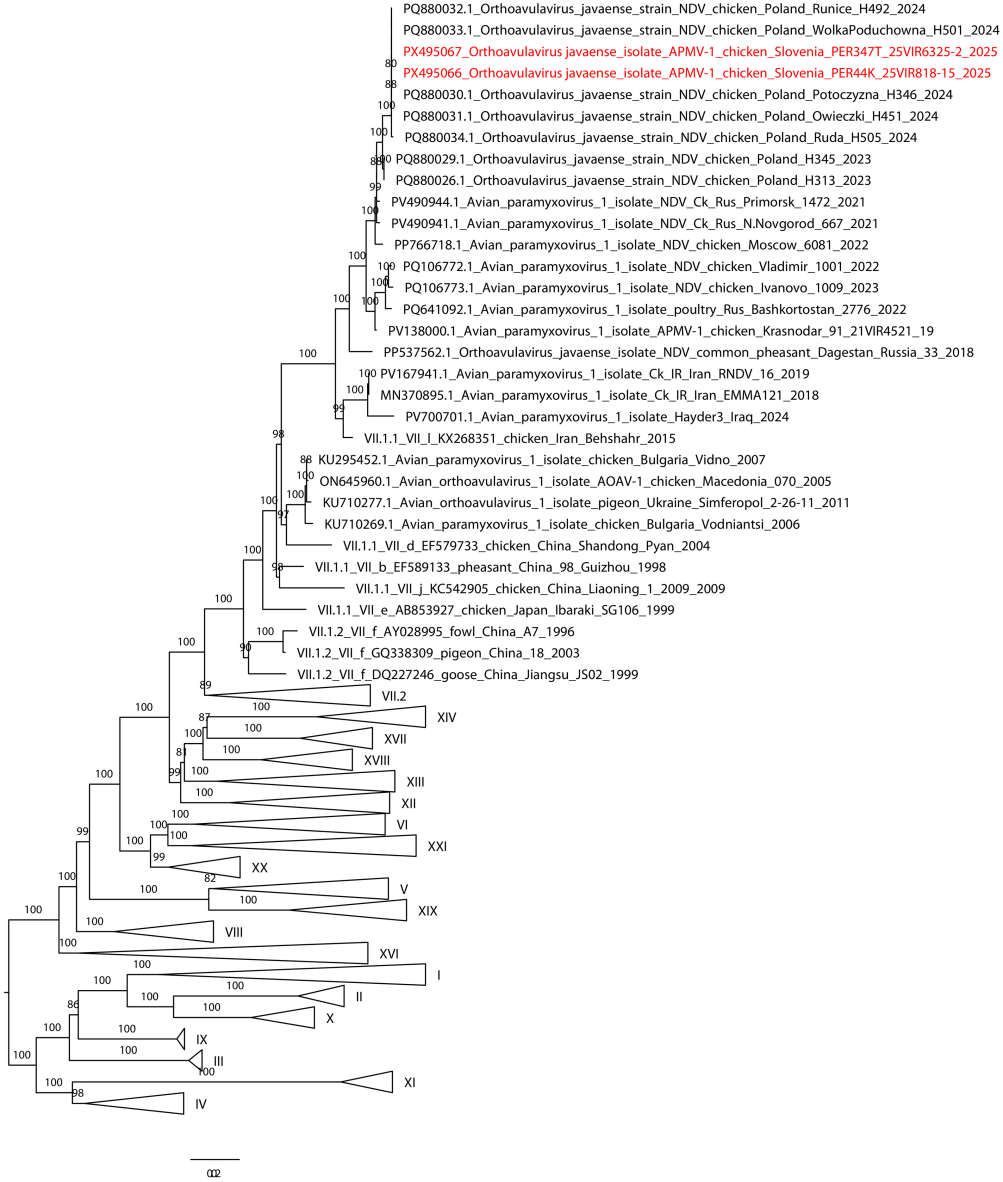
Maximum-likelihood phylogenetic tree based on the complete fusion (F) gene of class II OAVJ strains. Slovenian strains cluster within sub-genotype VII.1.1 together with viruses detected in Poland in 2023–2024, sharing 99.6–100% nucleotide identity. Bootstrap values above 80% are indicated at the nodes, and branch lengths correspond to the number of substitutions per site.

OAVJ was detected by real-time reverse transcription PCR (RT-PCR) for the L gene in cloacal, oropharyngeal and brain tissue samples collected during both outbreaks. All samples tested were negative for influenza A virus (IAV). Partial sequencing of the F gene revealed identical nucleotide sequences in samples from both outbreaks. These sequences showed 99% nucleotide identity with the OAVJ strain NDV/chicken/Poland/Potoczyzna/H346/2024 (GenBank accession number PQ880030.1) (see Figure 3). Analysis of the deduced amino acid sequence at the F protein cleavage site revealed presence of multiple basic amino acid residues at the C-terminus of the F2 protein with motif 112RRQKR116. The amino acid at the N-terminus of the F1 protein (residue 117) was deduced to be phenylalanine. The whole-genome sequences of OAVJ/chicken/Slovenia/25VIR818-15/2025 and OAVJ/chicken/Slovenia/25VIR6325-2/2025 is 15,160 nt in length and have been deposited in GenBank (Accession no. PX495066 and Accession no. PX495067). Phylogenetic analysis of the F gene places the Slovenian viruses within class II, sub-genotype VII.1.1; the two genomes share 100% nucleotide identity with each other and cluster with viruses detected in Poland in 2023–2024 (pairwise identity 99.6–100%).

The HN gene, which is critical for viral infection, also shows 100% nucleotide identity among the Slovenian strains. The HN glycoprotein comprises 571 amino acids, consistent with the typical length for genotype VII viruses. Several mutations with potential functional relevance were identified: substitutions E347Q and I514V in antigenic sites (potentially affecting HN antigenicity), and F156Y and T522I in sialic acid-binding site II, a region involved in sustaining contact with the target cell during fusion. In addition, an extra N-glycosylation motif is present at position 144 (Asn-Ile-Ser), which could influence viral pathogenicity (32). The functional significance of these substitutions in the genetic backgrounds analysed here remains to be determined.

## 4 Discussion

ND remains one of the biggest viral threats to global poultry production because it is highly contagious, has a broad host range and can cause severe economic losses. Despite decades of vaccination and control efforts, virulent OAVJ strains continue to circulate in many parts of the world, particularly in Asia, Africa and the Middle East, where the disease is endemic (1, 16). Sporadic outbreaks, often associated with backyard flocks or pigeon-adapted strains, highlight the ongoing threat of ND and its potential to spread rapidly if introduced into susceptible populations (14, 15).

In this study, we describe two outbreaks of ND in backyard and free-range chicken flocks in Slovenia in early 2025, marking the first confirmed cases in the country since 1991. The affected flocks exhibited characteristic clinical signs and gross lesions, including increases in mortality (57 and 73% in flocks 1 and 2, respectively), depression, cyanosis of the head, and haemorrhagic lesions in the gastrointestinal tract, which are consistent with the tissue tropism of viscerotropic velogenic strains and may reflect their capacity for systemic spread and potentially fatal disease (4). Histopathologic findings further supported the presence of velogenic OAVJ infection, as the observed lesion, including extensive necrosis and lymphocytic infiltration in multiple organs are consistent with the systemic spread and tissue tropism typical of highly virulent OAVJ strains (4, 16). In addition, the detection of secondary opportunistic pathogens such as *Escherichia coli*, *Clostridium perfringens*, *Pasteurella multocida*, and *Gallibacterium anatis* is likely a consequence of the immunosuppression and extensive epithelial damage caused by infection with a virulent OAVJ strain, as these effects facilitate bacterial invasion and proliferation, ultimately leading to increased morbidity and mortality (1, 4, 16). The presence of bacterial co-infections in both outbreaks emphasizes the importance of complete flock health management, as poor hygiene, parasite infestation and inadequate nutrition can aggravate disease severity, particularly in unvaccinated backyard flocks with limited veterinary management.

Molecular characterization and phylogenetic analysis revealed that both viruses belong to class II, genotype VII, subgenotype VII.1.1, a virulent lineage increasingly reported in Asia, Africa, the Middle East and Europe (6, 14–16). The Slovenian isolates clustered closely with VII.1.1 strains from Poland and Russia (18, 33), indicating a continuing dissemination of this lineage in the broader region. In Poland, reported ND outbreaks in recent years have occurred primarily in domestic poultry, particularly on commercial farms (34). Although detailed epidemiological investigations were not conducted in this study, the detection of closely related strains in different countries of the wider region (18, 33, 35) suggests the existence of an underlying epidemiological link. Nevertheless, the precise transmission pathways remain unknown.

In Slovenia, OAVJ s has been repeatedly detected in both feral and domestic pigeons as part of active and passive surveillance (21, 36) and they have mostly been assigned to genotype VI which is generally referred as pigeon paramyxoviruses 1 (PPMV-1). However, transmission to backyard or farm chickens has not been identified so far. In contrast, the current outbreaks in backyard/free-range chicken flocks were caused by velogenic OAVJ strains of subgenotype VII.1.1, suggesting that although pigeons can act as a reservoir for OAVJ, the viruses responsible for these outbreaks may have a distinct origin. OAVJ VII.1.1 was only detected in two backyard/free-range chicken flocks, neither of which was regularly vaccinated against ND. Despite the presence of the virulent virus in these two outbreaks, no further spread to commercial intensive poultry flocks, such as broilers, was observed. The lack of further spread in commercial poultry production could be due to the mandatory vaccination programs implemented in Slovenia for flocks with more than 350 birds, robust biosecurity practices in intensive broiler and layer farms (2, 37), and the prompt response of competent authorities and veterinary service in containing the outbreak. Regular vaccination of poultry has been shown to reduce both susceptibility to infection and virus shedding, thereby reducing the risk of onward transmission (1). In addition, strict biosecurity measures, including controlled access, hygiene protocols and separation from wild birds, are crucial components of ND prevention (2, 14, 15).

These results underline that despite the long-term absence of ND in Slovenia, local poultry populations remain highly susceptible to the introduction of virulent OAVJ, especially in unvaccinated backyard flocks without effective biosecurity measures.

## Data Availability

The original contributions presented in the study are publicly available. Sequence data has been deposited to GenBank, accession numbers PX495066 and PX495067.

## References

[1] Dimitrov KM Afonso CL Yu Q Miller PJ. Newcastle disease vaccines – a solved problem or a continuous challenge? Vet Microbiol. (2017) 206:126–36. doi: 10.1016/j.vetmic.2016.12.019, 28024856 PMC7131810

[2] World Organisation for Animal Health (WOAH). Manual of Diagnostic Tests and Vaccines for Terrestrial Animals. 13th ed. Paris: WOAH (2024).

[3] International Committee on Taxonomy of Viruses (ICTV). Virus taxonomy. Available online at: https://ictv.global/taxonomy/ (Accessed October 2, 2025)

[4] Alexander DJ. Newcastle disease and other avian paramyxoviruses. Rev Sci Tech Off Int Epizoot. (2000) 19:443–62. doi: 10.20506/rst.19.2.1231 10935273

[5] Morrison TG. Structure and function of a paramyxovirus fusion protein. Biochim Biophys Acta Biomembr. (2003) 1614:73–84. doi: 10.1016/S0005-2736(03)00164-0, 12873767

[6] Dimitrov KM Sharma P Volkening J Afonso CL Goraichuk IV Wajid A . Updated unified phylogenetic classification system and revised nomenclature for Newcastle disease virus. Infect Genet Evol. (2019) 74:103917. doi: 10.1016/j.meegid.2019.103917 31200111 PMC6876278

[7] Abolnik C Mubamba C Dautu G Gummow B. Complete genome sequence of a Newcastle disease genotype XIII virus isolated from indigenous chickens in Zambia. Genome Announc. (2017) 5:e00841–17. doi: 10.1128/genomeA.00841-17, 28839026 PMC5571412

[8] Barman LR Nooruzzaman M Sarker RD Rahman MT Saife MRB Giasuddin M . Phylogenetic analysis of Newcastle disease viruses from Bangladesh suggests continuing evolution of genotype XIII. Arch Virol. (2017) 162:3177–82. doi: 10.1007/s00705-017-3479-x, 28687921

[9] Sultan HA Elfeil WK Nour AA Tantawy L Kamel EG Eed EM . Efficacy of the Newcastle disease virus genotype VII.1.1-matched vaccines in commercial broilers. Vaccine. (2021) 10:29. doi: 10.3390/vaccines10010029, 35062690 PMC8779737

[10] Dharmayanti NI Nurjanah D Nuradji H Suyatno T Indriani R. Newcastle disease virus: the past and current situation in Indonesia. J Vet Sci. (2023) 25:e3. doi: 10.4142/jvs.23022, 38311318 PMC10839176

[11] Amoia CF Hakizimana JN Chengula AA Munir M Misinzo G Weger-Lucarelli J. Genomic diversity and geographic distribution of Newcastle disease virus genotypes in Africa: implications for diagnosis, vaccination, and regional collaboration. Viruses. (2024) 16:795. doi: 10.3390/v16050795, 38793675 PMC11125703

[12] Annaheim D Vogler BR Sigrist B Vögtlin A Hüssy D Breitler C . Screening of healthy feral pigeons (Columba livia domestica) in the city of Zurich reveals continuous circulation of pigeon paramyxovirus-1 and a serious threat of transmission to domestic poultry. Microorganisms. (2022) 10:1656. doi: 10.3390/microorganisms10081656, 36014074 PMC9412584

[13] Byrne AMP Mollett BC Brown IH James J Banyard AC Ross CS. Phylogenetic analysis of pigeon paramyxovirus type 1 (PPMV-1) detected in the British Isles between 1983–2023. Virus Evol. (2025) 11:veaf075. doi: 10.1093/ve/veaf075, 41089326 PMC12516948

[14] Department for Environment, Food & Rural Affairs; Animal and Plant Health Agency. Preliminary Outbreak Assessment #1: Newcastle disease (ND) in Poland and Europe, 6 June 2025. London: DEFRA/APH Agency. (2025). Available online at: https://assets.publishing.service.gov.uk/media/68519db8cf42a58f50cac9d8/6_June_2025_Newcastle_disease__ND__in_Poland_and_Europe.pdf (Accessed November 14, 2025).

[15] Department for Environment, Food & Rural Affairs; Animal and Plant Health Agency. Preliminary Outbreak Assessment #2: Newcastle disease (ND) in Spain, Poland and Europe, 26 January 2026. London: DEFRA/APH Agency; (2026). Available online at: https://assets.publishing.service.gov.uk/media/697cd2f2f0e5cf1ed2612d49/26_January_2026_Newcastle_disease_in_Spain__Poland_and_Europe.pdf (Accessed March 4, 2026).

[16] Miller PJ Koch G. "Newcastle disease". In: Swayne DE Boulianne M Logue CM McDougald LR Nair V Suarez DL, editors. Diseases of Poultry, 14th Edn. Hoboken, NJ: Wiley-Blackwell (2020). p. 89–138.

[17] Republika Slovenija. Pravilnik o ukrepih za ugotavljanje, preprečevanje in zatiranje atipične kokošje kuge. Uradni list RS. (2018) 81:12877–12883.

[18] World Organisation for Animal Health (WOAH). World Animal Health Information System (WAHIS): Animal Health Data Portal. Available online at: https://www.woah.org/en/what-we-do/animal-health-and-welfare/disease-data-collection/world-animal-health-information-system

[19] Spackman E Senne DA Myers TJ Bulaga LL Garber LP Perdue ML . Development of a real-time reverse transcriptase PCR assay for type a influenza virus and the avian H5 and H7 hemagglutinin subtypes. J Clin Microbiol. (2002) 40:3256–60. doi: 10.1128/JCM.40.9.3256-3260.2002, 12202562 PMC130722

[20] Sutton DA Allen DP Fuller CM Mayers J Mollett BC Löndt BZ . Development of an avian avulavirus 1 (AAvV-1) L-gene real-time RT-PCR assay using minor groove binding probes for application as a routine diagnostic tool. J Virol Methods. (2019) 265:9–14. doi: 10.1016/j.jviromet.2018.12.001, 30579921

[21] Krapež U Steyer AF Slavec B Barlič-Maganja D Dovč A Račnik J . Molecular characterization of avian paramyxovirus type 1 (Newcastle disease) viruses isolated from pigeons between 2000 and 2008 in Slovenia. Avian Dis. (2010) 54:1075–80. doi: 10.1637/9161-111709-ResNote.1, 20945791

[22] Di Tommaso P Chatzou M Floden EW Prieto Barja P Palumbo E Notredame C. Nextflow enables reproducible computational workflows. Nat Biotechnol. (2017) 35:316–9. doi: 10.1038/nbt.3820, 28398311

[23] Martin M. Cutadapt removes adapter sequences from high-throughput sequencing reads. EMBnet J. (2011) 17:10–2. doi: 10.14806/ej.17.1.200

[24] Andrews S. FastQC: A Quality Control Tool for High-Throughput Sequence Data. (2010). Available online at: http://www.bioinformatics.babraham.ac.uk/projects/fastqc (Accessed November 14, 2025).

[25] Li H. Aligning sequence reads, clone sequences and assembly contigs with BWA-MEM. arXiv. (2013). Available online at: https://arxiv.org/abs/1303.3997

[26] Van der Auwera GA O’Connor BD. Genomics in the Cloud: Using Docker, GATK, and WDL in Terra. 1st ed. Sebastopol: O’Reilly Media; (2020). Available online at: https://www.oreilly.com/library/view/genomics-in-the/9781491975183

[27] Wilm A Aw PPK Bertrand D Yeo GHT Ong SH Wong CH . LoFreq: a sequence-quality aware, ultra-sensitive variant caller for uncovering cell-population heterogeneity from high-throughput sequencing datasets. Nucleic Acids Res. (2012) 40:11189–201. doi: 10.1093/nar/gks918, 23066108 PMC3526318

[28] Kumar S Stecher G Tamura K. MEGA7: molecular evolutionary genetics analysis version 7.0 for bigger datasets. Mol Biol Evol. (2016) 33:1870–4. doi: 10.1093/molbev/msw054, 27004904 PMC8210823

[29] Katoh K Standley DM. MAFFT multiple sequence alignment software version 7: improvements in performance and usability. Mol Biol Evol. (2013) 30:772–80. doi: 10.1093/molbev/mst010, 23329690 PMC3603318

[30] Nguyen LT Schmidt HA von Haeseler A Minh BQ. IQ-TREE: a fast and effective stochastic algorithm for estimating maximum-likelihood phylogenies. Mol Biol Evol. (2015) 32:268–74. doi: 10.1093/molbev/msu300, 25371430 PMC4271533

[31] Rambaut A. (2010). FigTree v1.3.1. Institute of Evolutionary Biology, University of Edinburgh. Available online at: http://tree.bio.ed.ac.uk/software/figtree/

[32] Babaeimarzangou SS Allymehr M Molouki A Talebi A Fallah Mehrabadi MH. Identification of an additional N-glycosylation site and thermostable mutations within the hemagglutinin-neuraminidase gene of Newcastle disease virus belonging to sub-genotype VII.1.1. Vet Res Forum. (2023) 14:447–56. doi: 10.30466/vrf.2022.558074.3562, 37667791 PMC10475166

[33] Rtishchev A Treshchalina A Shustova E Boravleva E Gambaryan A. An outbreak of Newcastle disease virus in the Moscow region in the summer of 2022. Vet Sci. (2023) 10:404. doi: 10.3390/vetsci10060404, 37368790 PMC10304090

[34] European Commission. Newcastle disease outbreaks in Poland, first half of 2023. Directorate-General for Health and Food Safety, Brussels, Belgium; (2025). Available online at: https://food.ec.europa.eu/document/download/4560f359-fa07-4c77-a37c-b4853e12dac9_en?filename=reg-com_ahw_20250320_pres-20.pdf

[35] Guseva NA Kolosov SN Zinyakov NG Kozlov AA Shcherbakova LO Chvala IA . Subgenotype VII.1.1 Newcastle disease virus evolution and spread in the Russian Federation in 2019–2023. Viruses. (2025) 17:1319. doi: 10.3390/v17101319, 41157591 PMC12567687

[36] Dovč A Zorman-Rojs O Vergles Rataj A Bole-Hribovšek V Krapež U Dobeic M. Health status of free-living pigeons (Columba livia domestica) in the city of Ljubljana. Acta Vet Hung. (2004) 52:219–26. doi: 10.1556/AVet.52.2004.2.10, 15168753

[37] European Commission. Commission delegated regulation (EU) 2020/687 of 17 December 2019 supplementing regulation (EU) 2016/429 of the European Parliament and of the council as regards rules for the prevention and control of certain listed diseases. Off J Eur Union (2020) L174:64–142. Available online at: https://eur-lex.europa.eu/eli/reg_del/2020/687/oj

